# Revealing the effect of 2D carbides with different metal sites for improving hydrogen storage in MgH_2_

**DOI:** 10.3389/fchem.2022.1000408

**Published:** 2022-09-21

**Authors:** Kaixiang Ren, Bingbing Wang

**Affiliations:** School of Materials Science and Engineering, Anhui University of Technology, Maanshan, China

**Keywords:** hydrogen storage, MgH_2_, MXene, 2D materials, catalysis

## Abstract

Two-dimensional (2D) carbon materials are considered as efficient catalysts for improving hydrogen storage in MgH_2_, but their catalytic mechanisms of different materials remain unclear. Herein we compare the hydrogen storage properties of MgH_2_ with doping different 2D carbon materials for revealing their catalytic effecting mechanisms. It can be seen that the effect of 2D metal carbides including Nb_2_C and Ti_2_C are superior to 2D graphene for improving hydrogen storage properties of MgH_2_, where the Ti_2_C exhibits the best catalytic effect with a remarkable decrease of activation energy (*E*_a_) from ∼124 kJ/mol for doping graphene to ∼86 kJ/mol. This is related to the changes of individual metal and graphite chemical valence states of catalysts. The high catalytic activity of the hydrogen storage reaction originates from its unique layered structure and *in situ* formation of MH_X_, i.e., the tiny metal crystals can serve as a channel to facilitate hydrogen transport in MgH_2_ matrix. Moreover, the Ti catalytic effect is better than Nb, which originates from the surface of the multivalent Ti atoms is an intermediate of the electron moving between H^−^ and Mg^2+^, thus leading to the Ti_2_C catalyzed MgH_2_ with superior hydrogen kinetic and cyclic performance.

## Introduction

Magnesium ranks sixth in the Earth’s crust, is widely distributed in nature, is inexpensive, and its 7.6 wt% hydrogen storage capacity and good reversibility make magnesium a hot topic for hydrogen storage materials (Nabgan et al., 2017; Shao et al., 2018). Meanwhile, the position of magnesium in the periodic table is the second main family in the third cycle, belonging to lively metal. Thus, magnesium oxide films will form on the surface, which is not conducive to the hydrogenation reaction. Besides, the hydrogenation enthalpy of MgH_2_ was 76.0 kJ/mol. It is much higher than that of most metal hydrides (generally 20–40 kJ/mol), resulting in poor thermodynamic stability, which is due to the strong ionic bond between hydrogen and magnesium (Ding et al., 2017; Si et al., 2021); finally, MgH_2_ starts to absorb hydrogen at about 285°C and hydrogenates at 385°C, because the molecular hydrogen needs to overcome 432 kJ/mol energy barrier to carry out the hydrogenation reaction with magnesium (Zhang et al., 2011). The high temperature of hydrogen absorption and desorption and the slow rate of hydrogen absorption and release lead to its poor kinetic performance (Chen and Zhu, 2008; Jain et al., 2010; Ma et al., 2017). Although MgH_2_ has certain advantages in hydrogen storage materials, it still needs to be further improved in its hydrogen storage performance to achieve practical application.

Lowering the operating temperature and increasing the rate of dehydrogenation of MgH_2_ are key challenges for its application, which can be overcome by catalytic, alloying, nano-grading and nano-constraining methods (Xia et al., 2015; Yu et al., 2017; Zhu et al., 2020a). Adjusting the binding energy of the Mg-H bonds by the alloy element (Edalati et al., 2018) or locally by additives is an effective way to alter the MgH_2_ thermodynamics. Catalyst doping (Wang et al., 2018) has proven to be the most feasible and efficient method to accelerate MgH_2_ dehydrogenation dynamics. However, nanoscale Mg-based hydrogen storage materials will agglomerate after repeated hydrogen absorption and discharge, leading to the degradation of hydrogen storage performance, and similarly repeated Mg-based alloy-based hydrogen storage material powders will lead to the degradation of hydrogen storage performance. It was shown that the hydrogen adsorption performance of mixtures of MgH_2_ and carbon materials including graphite, activated carbon, multi-walled carbon nanotubes (MWCNTs), carbon nanofibers (CNF) and activated carbon fibers were studied by M. A. et al. (Lillo-Ródenas et al., 2008). By comparison, the introduction of graphite can effectively reduce the decomposition temperature of MgH_2_. This is due to the superior physicochemical properties and high specific surface area of graphene, and most importantly, graphene has a two-dimensional monolayer crystal structure, which increases the interfacial area and thus provides more active catalytic sites (Novoselov et al., 2004).

Moreover, transition metal (TM) [Ni (Zou et al., 2012), Ti (Ren et al., 2014; Lotoskyy et al., 2018), Nb (Liu et al., 2015), Fe (Chen et al., 2016), Co (Lu et al., 2017; Khan et al., 2019)], transition metal oxides [TiO_2_ (Zhang et al., 2019; Ma et al., 2020), Nb_2_O_5_ (Hanada et al., 2006)] and transition metal-based alloys [TiCu (Zhou et al., 2019), FeNb (Santos et al., 2014)] are the most feasible and reliable alloys for the catalysts of magnesium-based hydrogen storage materials. Meanwhile, two-dimensional carbon material catalysts are also widely used in MgH_2_ systems such as Ti_3_C_2_/TiO_2_(A)-C (Gao et al., 2020), Ni@FL-Ti_3_C_2_ (Zhu et al., 2020b; Gao et al., 2020; ), Ni@C-Mxene (Huang et al., 2021), etc. Large surface area or nanobunching can explain the excellent catalytic efficiency of catalysts with unique structural features. Ma et al. (2013) reported the distribution of small metal nanocrystals in the composites as a way to facilitate hydrogen transport and improve dehydrogenation performance. A contradictive mechanism was later proposed by Li et al. (2006) in density flooding theory calculations, and their analysis showed that Ti_2_C is only a catalyst rather than a reactant, where the polyvalent Ti atoms on the surface act as an intermediate for electron transport between H^−^ and Mg^2+^, making dehydrogenation easier. And the good hydrogen adsorption capacity and thermal conductivity of Ti_2_C 2D materials also contribute to the improvement of the dehydrogenation thermodynamics of MgH_2_. These contradictive mechanisms enable us to further clarify the intrinsic catalytic mechanism by comparison of different 2D materials including graphene, Nb_2_C, and Ti_2_C.

In this paper, the distinct hydrogen storage properties of Mg-based materials are examined by adding different 2D carbides catalysts. As compared to single graphene catalysts, the synergy of introduced transition metal TM (Nb, Ti) and graphite was systematically studied to explore the effect of each catalyst on the hydrogen storage performance of MgH_2_, and the catalytic effect of two composites including MgH_2_@Nb_2_C and MgH_2_@Ti_2_C was carefully compared with MgH_2_@Graphene, thus revealing the difference in the effect of 2D carbides on the hydrogen storage of MgH_2_.

## Experimental section

### Preparation of MgH_2_ 2D carbon composites

The starting chemicals of magnesium hydride (MgH_2_, purity 95%, powder) and two-dimensional graphene (Graphene, purity 99%, powder) from Alfa Aesar and Nb_2_C (purity 99%, powder) and Ti_2_C (purity 98%, powder) from Xianfeng Nanomaterials Technology Co, China. First, a total of 500 mg of MgH_2_ powder and 2D graphene powder with a mass ratio of 19:1 were weighed and placed in a ball mill jar with an argon atmosphere, a ball-to-material ratio of 40:1, a rotation speed of 400 r/min, and ball milled on a ball mill model XQM-2A for 10 h. The treatment of MgH_2_ as a comparison sample and the process of preparing MgH_2_@Graphene was kept consistent. Then, the preparation process of MgH_2_@Nb_2_C and MgH_2_@Ti_2_C was kept the same as that of preparing MgH_2_@Graphene.

### Characterization and hydrogen sorption measurements

To understand the composition and structure of the samples, the properties of the samples such as cell parameters, crystallographic surface indices, dot matrix parameters and atomic occupancies were further analyzed using software such as Jade 6 and Powder X, respectively. The XRD instrument used to test the samples was a D2 PHASER XE-T Edition with an energy resolution of up to 380 eV, and a Cu target was selected as the ray tube. The hydrogen desorption capacity, temperature and reaction rate of the hydrogen storage material were measured directly using DSC. For the experiments, a 20 mg sample is placed in a quartz vessel and then placed in a reaction vessel at a temperature range from room temperature to 400°C, with the heating rate set by the properties of the sample. During the test, the mechanical automatic recording of the corresponding mass and temperature at each temperature can further calculate the amount of hydrogen release, heat absorption peak and heat release peak. To further observe the tissue morphology of the composites, a scanning electron microscope (SEM) of NONA NanoSEM 430 from DYC was used to observe the composites and combined with Mapping to analyze and determine the elemental composition and elemental distribution. Before sample testing, the samples were treated with conductive adhesive and observed using backscattered electron imaging mode. Talos F 200 X S/TEM type equipment was used to further visualize the tissue morphology of the samples through microscopic (TEM) and high resolution (STEM) images, and then combined with Digital Micrograph software to perform IFFT (Fast Fourier Transform) and FFT (Fast Fourier Transform) on the transmission electron diffraction images of the samples to observe the lattice images, atomic arrangement and crystallographic indices. To prepare the samples, they were placed in tetrahydrofuran (THF), followed by ultrasonic shaking, well dispersed and coated onto porous carbon films, and finally dried. All preparations are done in a glove box containing argon gas (>99.99% purity). The samples required for the experiment are loaded into the reaction vessel and then taken out of the glove box and connected to the P-C-T experimental setup to enter the testing phase. In the constant temperature hydrogen absorption and discharge test phase, the amount of hydrogen released can be found according to the pressure change and the curve of hydrogen pressure change with time can be made.

## Results and discussion

### Structural and morphological features

**FIGURE 1:**
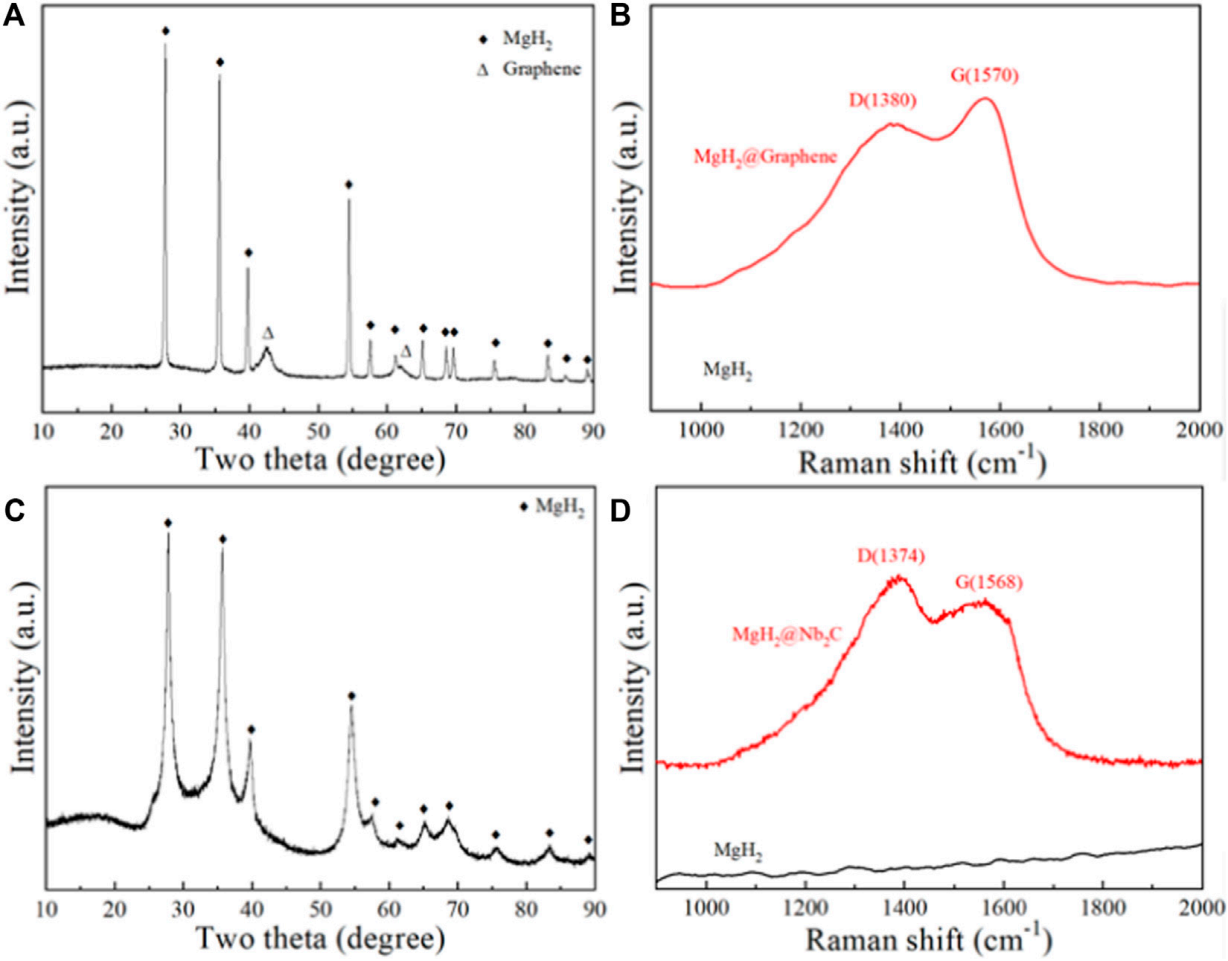
MgH_2_@Graphene composites for **(A)** XRD pattern and **(B)** Raman pattern, MgH_2_@Nb_2_C composites for **(C)** XRD pattern and **(D)** Raman pattern.

Figure 1A shows the XRD pattern of the ball-milled product of MgH_2_@Graphene composite, from which the diffraction peaks of MgH_2_ and Graphene can be seen, indicating that the graphene is loaded with MgH_2_. To further prove that the ball-milled product contains Graphene, the Raman test was performed on it, and the Raman pattern is shown in Figure 1B, with wave numbers 1,380 and 1,570 cm^−1^ show distinct peaks, which represent the D-band and G-band of graphene, respectively; the D-band corresponds to disordered carbon or defective graphite structures in the sample, and the G-band is characteristic of graphite layers (Grochala and Edwards, 2004), confirming the presence of graphene. Figure 1C shows the XRD image of the ball-milled product of the MgH_2_@Nb_2_C composite, from which only MgH_2_ is present in the ball-milled product, probably because the carbon and niobium nanosized particles are too fine to be detected, to further prove the presence of carbon and niobium in the ball-milled product, Raman tests were performed on it. The Raman spectrum is shown in Figure 1D, which shows distinct peaks at wave numbers 1,374 and 1,568 cm^−1^, which represent the D-band and G-band of graphene, respectively, confirming the carbon as graphene.

**FIGURE 2:**
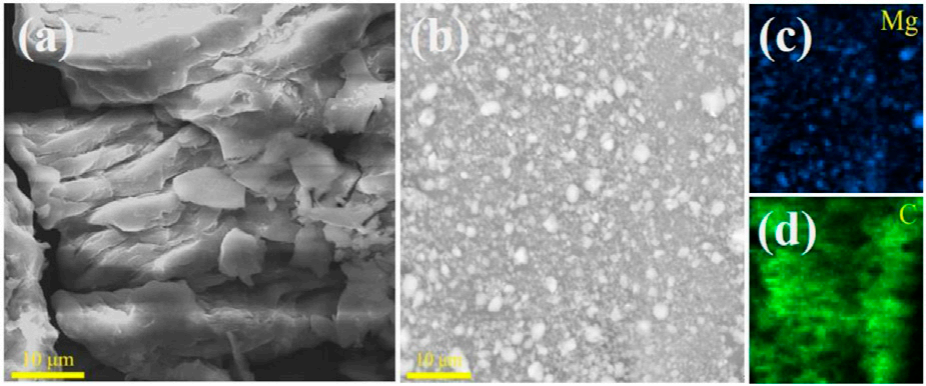
SEM images for **(A)** MgH_2_, **(B)** MgH_2_@Graphene, and **(C,D)** corresponding elemental mapping images of Mg and **(C)**.

To further study the microscopic morphology of the MgH_2_@Graphene and MgH_2_@Nb_2_C composites, scanning electron microscopy analysis was performed. The scanning electron microscopy analysis of MgH_2_@Graphene composites is shown in Figure 2. It is obvious that the MgH_2_@Graphene particles are smaller than those of pure MgH_2_, and it can also be found that the MgH_2_@Graphene particles are more uniformly distributed in size and do not appear to be agglomerated; the corresponding elemental distribution diagram observes that the carbon is more like a mesh structure, making the MgH_2_ particles uniformly loaded on the graphite layer. The finer MgH_2_@Graphene particles are attributed to the reaction process between MgH_2_ and two-dimensional graphene. On the one hand, graphene provides a reaction attachment point for the nucleation growth of magnesium grains, and on the other hand, graphene restricts the MgH_2_@Graphene particles. Meanwhile, graphene restricts the mobility of MgH_2_ grains, making them difficult to agglomerate, thus causing the refinement of MgH_2_ grains while inhibiting the agglomeration of MgH_2_.

**FIGURE 3:**
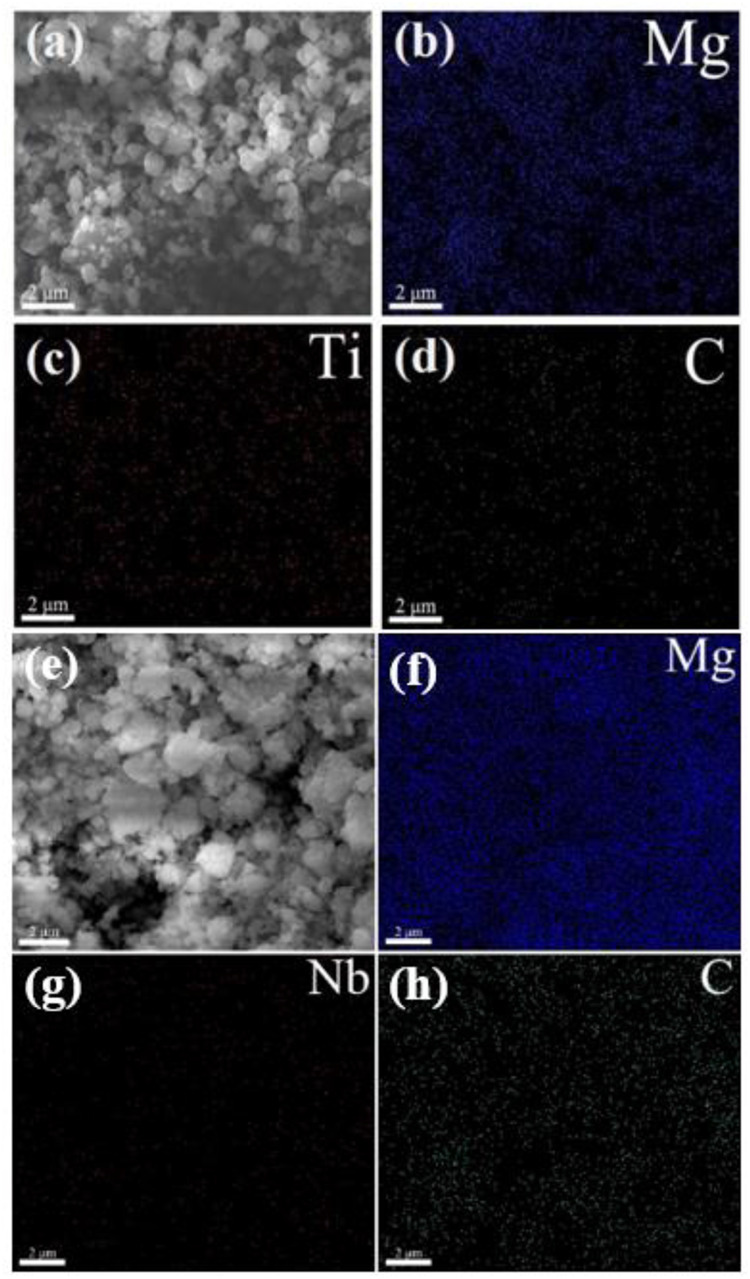
MgH_2_@Ti_2_C **(A–D)** and MgH_2_@Nb_2_C **(E–H)** SEM pattern of composites.

The scanning electron microscopy analysis of the MgH_2_@Ti_2_C composite is shown in Figures 3A–D, and the scanning electron microscopy analysis of the MgH_2_@Nb_2_C composite is shown in Figures 3E–H. The presence of Ti and Nb elements is visible at a magnification of ×5,000, confirming the presence of the transition metal Ti as well as the metal Niobium. The SEM images also reveal a more uniform distribution of the composite particle sizes without agglomeration, and the corresponding elemental distribution maps indicate a homogeneous distribution of both Ti and C as well as Nb and C on the Mg substrate. In summary, the introduction of graphene-based transition metals Ti and Nb is feasible and the MgH_2_@Ti_2_C and MgH_2_@Nb_2_C composites were successfully prepared.

### Improved hydrogen storage properties

**FIGURE 4:**
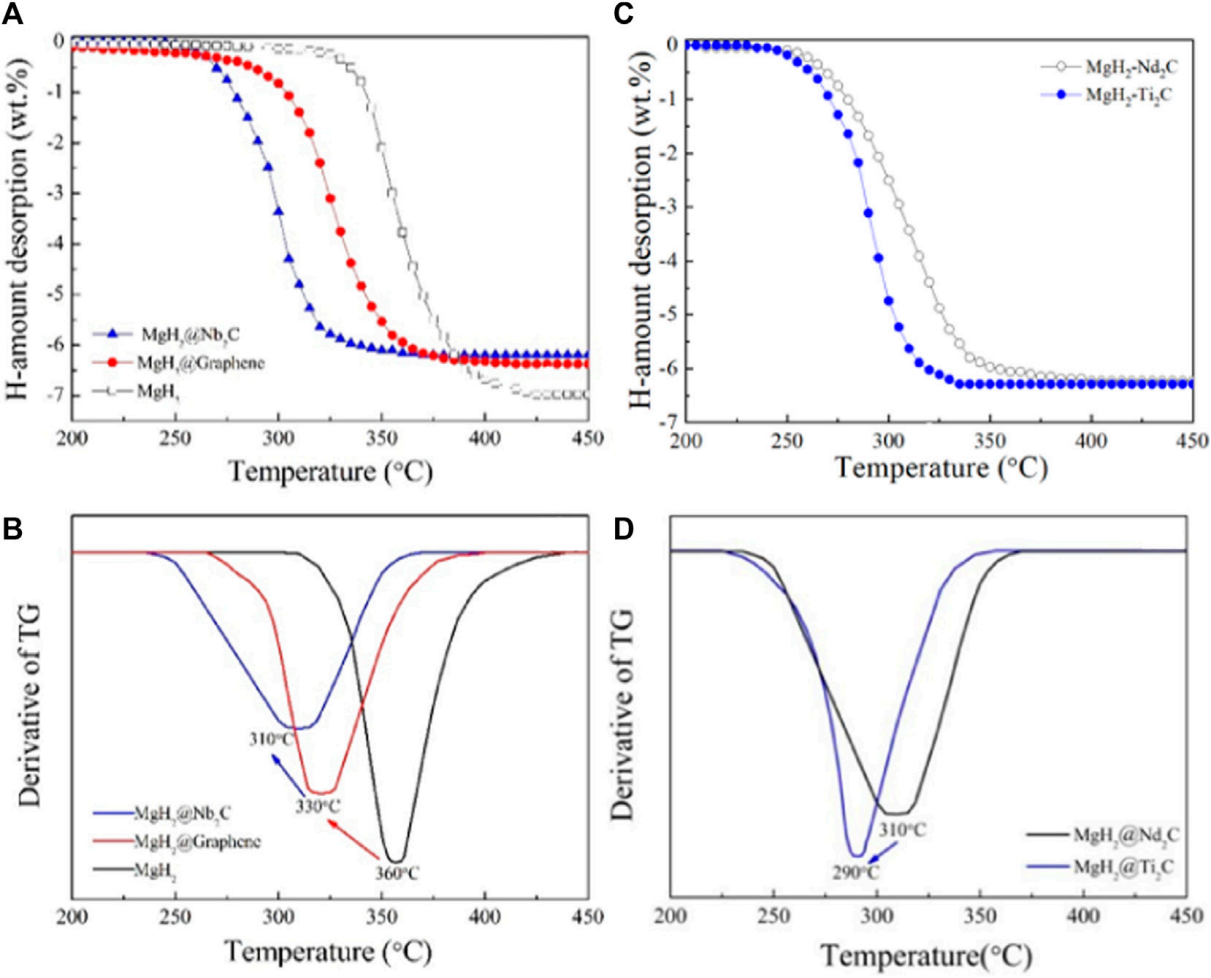
**(A)** The temperature—programmed desorption curves for the MgH_2_@Nb_2_C **(B)** corresponding first order derivative graph, **(C)** The temperature-programmed desorption curves for the MgH_2_@Ti_2_C **(D)** corresponding first order derivative graph.

Figure 4A shows the temperature-programmed desorption curves of MgH_2_@Nb_2_C composite, MgH_2_@Graphene composite and pure MgH_2_, from which the starting hydrogen release temperature of pure MgH_2_ is 305°C and the ending hydrogen release temperature is 425°C, while the starting hydrogen release temperature of MgH_2_@Graphene composite prepared by mechanical ball milling is 260°C and the ending hydrogen release temperature is 405°C. To derive the minimum temperature at which the maximum hydrogen release rate is achieved, we differentiate Figure 4A to obtain Figure 4B, from which we know that the minimum temperature at which the maximum hydrogen release rate of pure MgH_2_ is 360°C, the minimum temperature of the maximum hydrogen release rate of MgH_2_@Graphene composite is at 330°C, while the MgH_2_@Nb_2_C composite is 310°C, which is 50°C lower than that of pure MgH_2_, indicating that the addition of transition metal niobium on top of graphene can effectively reduce the temperature of the maximum hydrogen release rate. In summary, the addition of the two-dimensional layered metal carbide Nb_2_C can further reduce the hydrogen release temperature of pure MgH_2_ compared to that of single graphene. The two-dimensional layered metal carbide Nb_2_C has a remarkable catalytic effect on MgH_2_ hydrogen storage properties. To further validate the catalytic effect of two-dimensional carbon materials with different metal sites on improving the hydrogen storage of MgH_2_, we chose the two-dimensional layered metal carbide Ti_2_C as the catalyst for MgH_2_. Figure 4C shows the temperature-programmed desorption curves of MgH_2_@Ti_2_C and MgH_2_@Nb_2_C composites. It can be seen from the figure that the initial desorption temperature of the MgH_2_@Nb_2_C composite is 250°C and the termination temperature is 350°C. The initial desorption temperature of MgH_2_@Ti_2_C is 235°C and the termination temperature is 325°C, indicating that under the same experimental conditions, the lower desorption temperature of MgH_2_@Ti_2_C and the better catalytic performance of Ti_2_C. To obtain the minimum temperature of the maximum discharge rate, we differentiated Figure 4C and obtained Figure 4D. From Figure 4D, the minimum temperature of MgH_2_@Nb_2_C composite is 310°C, and 290°C for MgH_2_@Ti_2_C composite, the minimum temperature of the maximum discharge rate is reduced by 20°C, indicating that MgH_2_@Ti_2_C has faster hydrogenation rate, and the catalytic performance is better. In conclusion, the 2-dimensional layered metal carbide Ti_2_C can further reduce the hydrogenation temperature of MgH_2_ and make its discharge rate faster than the single graphene.

**FIGURE 5:**
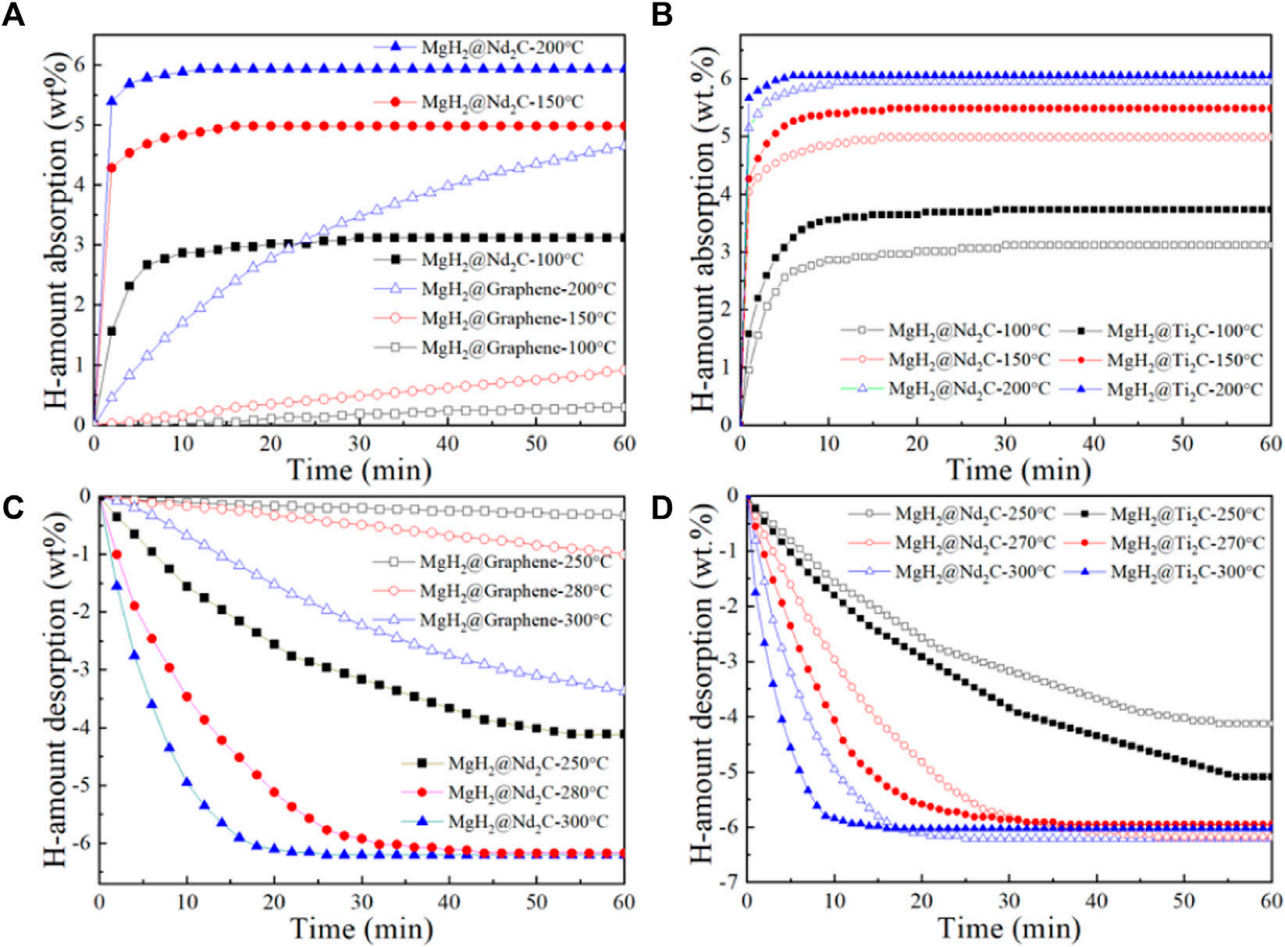
**(A)** MgH_2_@Nb_2_C, **(B)** MgH_2_@Ti_2_C isothermal hydrogen absorption curves at different temperatures, **(C)** MgH_2_@Nb_2_C, MgH_2_@Ti_2_C **(D)** Isothermal hydrogen desorption curves at different temperatures.

Figure 5A shows the isothermal hydrogen absorption curves of MgH_2_@Nb_2_C composites and MgH_2_@Graphene composites at different temperatures. It can be seen from the figure that MgH_2_ with Graphene addition has almost no hydrogen absorption behavior at 100°C and only 1 wt% at 150°C for 60 min. In contrast, MgH_2_@Nb_2_C composites, at 100°C for 20 min, absorb up to 3 wt% of hydrogen, which is much higher than MgH_2_@Graphene. Not only that, the hydrogen absorption of MgH_2_@Nb_2_C composite at 150°C is much higher than that of MgH_2_@Graphene. MgH_2_@Nb_2_C composites showed a higher hydrogen uptake rate and hydrogen uptake at 150°C than MgH_2_@Graphene composites at 200°C. It shows that the addition of two-dimensional layered metal carbide Nb_2_C leads to the better catalytic performance of MgH_2_ in terms of hydrogen uptake rate and hydrogen uptake capacity compared with the addition of graphene only. Figure 5B shows the isothermal hydrogen absorption curves at different temperatures for both the MgH_2_@Ti_2_C composite and the MgH_2_@Nb_2_C composite. It can be seen from the figure that the hydrogen uptake of MgH_2_@Ti_2_C composites and MgH_2_@Nb_2_C composites remained almost the same at 200°C for 60 min, but the hydrogen uptake rate of MgH_2_@Ti_2_C was somewhat faster; but at 150°C and 100°C, MgH_2_@Ti_2_C is not only faster and has higher hydrogen absorption capacity than MgH_2_@Nb_2_C.The isothermal hydrogen absorption properties of the MgH_2_@Ti_2_C composite material and the MgH_2_@Nb_2_C composites can differ significantly at different temperatures. Figure 5C shows the isothermal hydrogen release test of MgH_2_@Nb_2_C composite and MgH_2_@Graphene composite at different temperatures. It can be seen from the figure, the MgH_2_@Graphene composite has almost no hydrogen discharge behavior at 250°C, only 1 wt% at 280°C and 60 min, and only 3.4 wt% at 300°C and 60 mi. Relatively speaking, MgH_2_@ Nb_2_C composite has reached 4 wt% in 250°C and 50 min, which has far exceeded MgH_2_@Graphene in the discharge rate and amount. At 280°C and 300°C, MgH_2_@Nb_2_C composite is the same, reaching 6.4 wt%, However, the rate of hydrogen release accelerates substantially with increasing temperature. It is shown that the addition of the 2D layered metal carbide Nb_2_C improves MgH_2_ at the release rate and release capacity compared to only 2D graphene. Similarly, we conducted isothermal hydrogen desorption tests on MgH_2_@Nb_2_C and MgH_2_@Ti_2_C composites at different temperatures. The results are shown in Figure 5D. It can be seen from the figure that, within 60 min at 270°C and 300°C, the hydrogen emission amount of MgH_2_@Ti_2_C and MgH_2_@Nb_2_C is almost the same, but the hydrogen emission rate of MgH_2_@Ti_2_C is faster than that of MgH_2_@Nb_2_C. However, at 250°C, MgH_2_@Ti_2_C has a higher dehydrogenation rate and capacity than MgH_2_@Nb_2_C. The isothermal dehydrogenation properties of MgH_2_@Nb_2_C and MgH_2_@Ti_2_C are different at different temperatures.

**FIGURE 6:**
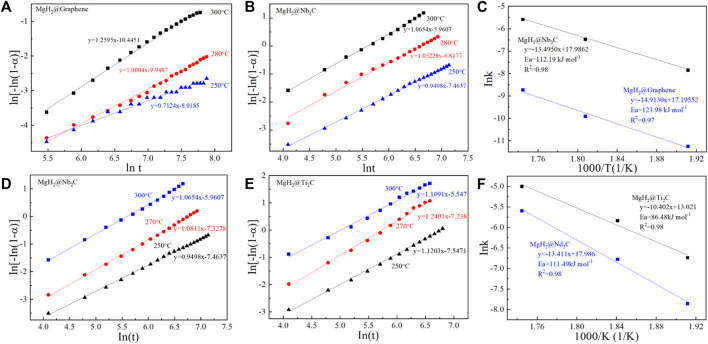
JMAK plots for the hydrogen desorption at different temperature **(A)** MgH_2_@Graphene, **(B)** MgH_2_@Nb_2_C and **(C)** corresponding Arrhenius plots, JMAK plots for the hydrogen desorption at different temperature **(D)** MgH_2_@Nb_2_C, **(E)** MgH_2_@Ti_2_C, and **(F)** corresponding Arrhenius plots.

To verify the advantages of MgH_2_@Ti_2_C, MgH_2_@Nb_2_C, and MgH_2_@Graphene in terms of kinetic performance, we also used the JMAK (*Johnson−Mehl−Avrami−Kolmogorov*) model (Shahi et al., 2014) to calculate their apparent hydrogen release activation energies. Based on the JMAK model, the desorption kinetics can be expressed by the following equation:

ln[−ln(1−α)]=ηlnk+ηlnt (1)

where *α* is the reaction fraction corresponding to the beginning and completion of the reaction, *η* is the *Avrami* index of the order of the reaction, *k* is the rate constant, and *t* is time. Sample data for experiment, ln [−ln (1−*α*)] as a function of ln(*t*) figure in different temperature of each curve is linear, as shown in Figures 6A,B,D,E. After calculating the rate constant k, the apparent activation energy (*E*_a_) of the dehydrogenation process is calculated according to the Arrhenius equation:

$$k=k_{0}\text{exp}\left(-E_{a}/RT\right)$$ (2)

where *k*_0_ is pre-exponential factor, *R* is gas constant and *T* is absolute temperature. From the slopes of the straight lines as shown in Figures 6C,F. The *E*_a_ of MgH_2_@Nb_2_C is 112.19 kJ/mol, 11.79 kJ/mol lower than that of MgH_2_@Graphene (123.98 kJ/mol). The calculated *E*_a_ of MgH_2_@Nb_2_C is 111.49 kJ/mol, and the *E*_a_ of MgH_2_@Ti_2_C was 86.48 kJ/mol, decreasing by 25.01 kJ/mol. The results indicate that the two-dimensional layered metal carbides Ti_2_C and Nb_2_C reduce the apparent activation energy of MgH_2_ compared with the addition of only two-dimensional graphene. More importantly, the 2D carbon materials with different metal sites improve the hydrogen storage of MgH_2_ catalytic effects showed significant differences.

**FIGURE 7:**
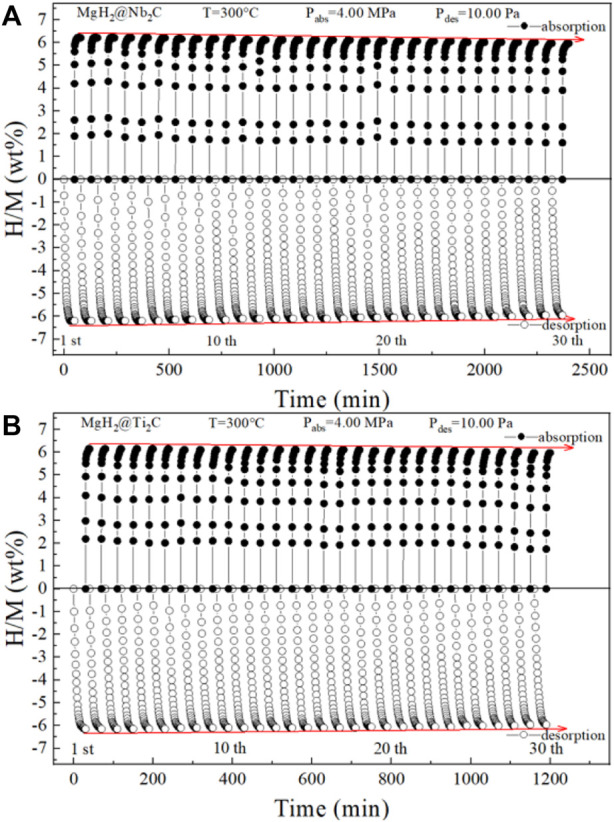
**(A)** MgH_2_@Nb_2_C **(B)** MgH_2_@Ti_2_C isothermal hydrogen absorption and desorption cycle curve.

Figures 7A,B show the isothermal hydrogen absorption and discharge cycle curves for MgH_2_@Nb_2_C and MgH_2_@Ti_2_C, where the hydrogen absorption temperature is 300°C and the hydrogen absorption pressure is 4 MPa, and the hydrogen discharge temperature is 300°C and the hydrogen discharge pressure is 10 Pa. The initial hydrogen discharge is 6.41 wt%, 6.39 wt%, and the final hydrogen absorption is 6.09 wt% and 6.19 wt%, respectively. The capacity retention was as high as 95% and 97% in isothermal hydrogen absorption and discharge cycles, respectively, with almost no decay, indicating that both MgH_2_@Nb_2_C and MgH_2_@Ti_2_C composites have good stability. The comparison of the two shows that the catalytic effect of transition metals and graphene on MgH_2_ is synergistic, and the catalytic effect of Ti is better than that of Nb, which makes the hydrogen uptake and discharge and kinetic performance of MgH_2_@Ti_2_C better than that of MgH_2_@Nb_2_C.

### Difference in catalytic mechanisms of 2D carbon materials

**FIGURE 8:**
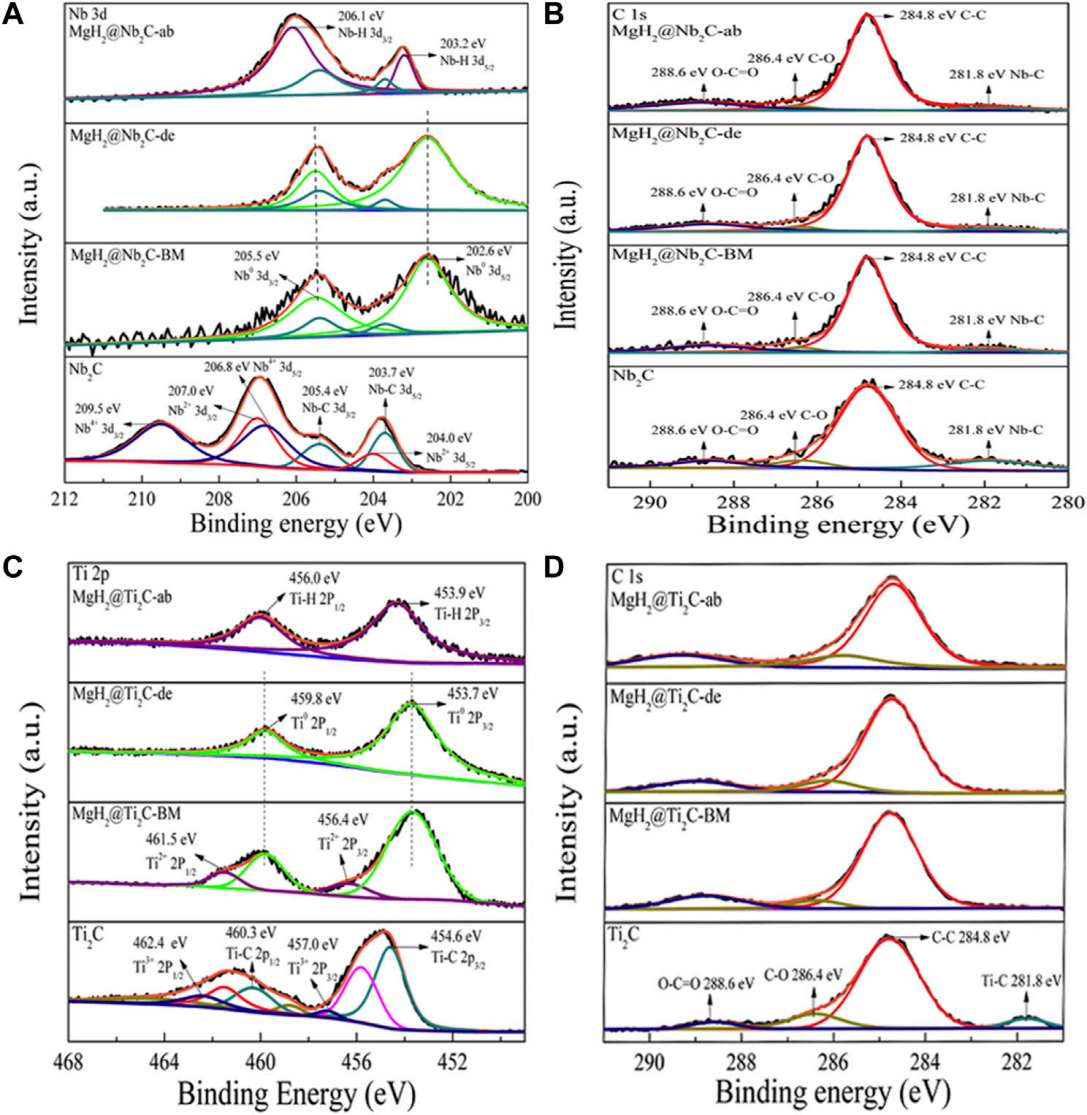
**(A)** Nb 3d and **(B)** C 1s XPS spectra of Nb_2_C and MgH_2_@ Nb_2_C samples at different states, **(C)** Ti 2p and **(D)** C 1s XPS spectra of Ti_2_C and MgH_2_@ Ti_2_C samples at different states.

The chemical valence state changes of Ti and C as well as Nb and C in the process of hydrogen absorption and release can be further analyzed by XPS. Figure 8A shows the XPS spectrum of Nb 3d with six peaks of 203.7/205.4, 204.0/207.0, and 206.8/209 eV for catalyst Nb_2_C. After the ball milling reaction with MgH_2_, it was found that both Nb^2+^ and Nb^4+^ peaks disappeared, Nb-C failed to disappear completely, and a new peak Nb^0^ was generated (corresponding to 202.6/205.5 eV), which indicates that the catalyst Nb_2_C produced metallic Nb during the high-energy ball milling process. However, the chemical price of the MgH_2_@Nb_2_C composite did not change during the first hydrogenation of Nb, but after the first hydrogen uptake, the Nb^0^ peak (corresponding to 202.6/205.5 eV) disappeared and a new Nb-H peak (corresponding to 203.2/206.1 eV) appeared. This indicates that the MgH_2_@Nb_2_C composite is hydrogenated by metal Nb to NbH_X_ after the first hydrogen uptake. Similarly, Figure 8C shows the XPS spectrum of Ti 2p, which also illustrates that the MgH_2_@Ti_2_C composite is hydrogenated by metal Ti to TiH_X_ after the first hydrogen uptake. Figures 8B,D shows the C 1s XPS spectrum. The results show that between the Ti_2_C and MgH_2_@Ti_2_C composites in different states and between the Nb_2_C and MgH_2_@Nb_2_C composites, only the Nb-C bond is broken with Ti-C and there is no change in C-C, indicating that the carbon layer is not decomposed. Investigation of the periodic table of elements shows that the electronegativity of Mg is ∼1.31 and that of H is ∼2.2, while the electronegativity of Ti is ∼1.54 and that of Nb is ∼1.60, between Mg and H, which can effectively weaken the Mg-H bond. In summary, after the reaction decomposition of catalyst Ti_2_C as well as Nb_2_C with MgH_2_, the zero-valent titanium/niobium grows *in situ* in Mg grains, and the carbon layer without decomposition will further form graphene, which can provide nucleation and growth of nano-MgH_2_, making the distribution of nano-MgH_2_ grains relatively uniform and effectively inhibiting the growth and agglomeration of MgH_2_ grains in the hydrogen cycle.

**FIGURE 9:**
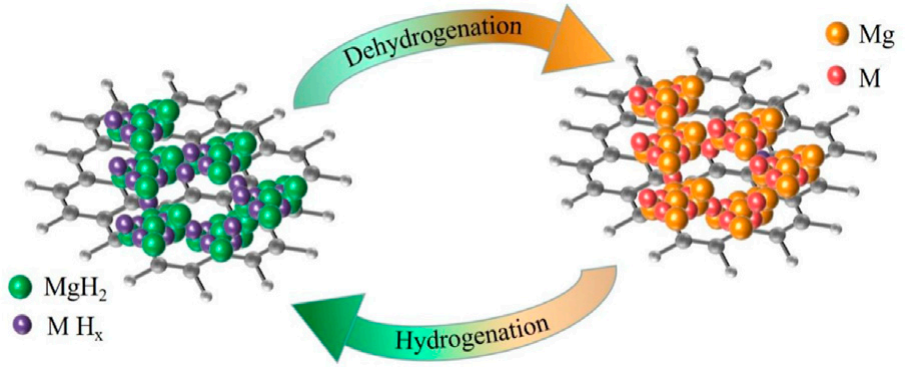
MgH_2_@*M*_2_C theoretical schematic diagram in the state of complete hydrogen absorption and complete hydrogen release.

To further explain the changes in the chemical composition of each metal and graphite during the hydrogen uptake and release process of MgH_2_@M_2_C (M is metal) composites, thus revealing the catalytic effect of 2D carbon materials with different metal sites on improving the hydrogen storage of MgH_2_, we combined XPS to map the flow of the materials in the hydrogen uptake and release stage. As shown in Figure 9, hydrogen stage, weakened MgH_2_ will preferentially decompose into magnesium grains and free hydrogen atoms, free hydrogen atoms on the MH_X_ surface reunited into H_2_ molecules, then MH_X_ will decompose into metal Nb and hydrogen atoms, also hydrogen atoms will be combined into H_2_ molecules. In contrast, in the hydrogen absorption phase, metal Nb first reacts with H_2_ to form MH_X_, the H_2_ molecule dissociates into hydrogen atoms on the surface of MH_X_, free hydrogen atoms react with magnesium through the niobium supply channel to form magnesium hydrogen bonds, and the formed MgH_2_ meeting further nucleates and grows into MgH_2_ grains.

## Conclusion

In this paper, MgH_2_@Graphene, MgH_2_@Nb_2_C and MgH_2_@Ti_2_C composites were successfully prepared by ball milling reaction of two-dimensional layered metal carbides Nb_2_C and Ti_2_C with MgH_2_, and their hydrogen storage properties were tested. The results show that the apparent activation energy of MgH_2_@Nb_2_C composite is ∼111.49 kJ/mol, and that of MgH_2_@Ti_2_C composite is ∼86.48 kJ/mol, which is lower than ∼123.98 kJ/mol of MgH_2_@Graphene composite. In 30 cycles of hydrogen absorption and desorption, the hydrogen desorption capacity of MgH_2_@Ti_2_C composite is as high as 97.0%, and that of MgH_2_@Nb_2_C composite is as high as 95.0%, indicating that the addition of Ti_2_C has lower apparent activation energy and better cycle stability than that of Nb_2_C. The tiny M (Ti/Nb) crystals can act as a channel to facilitate hydrogen transport and improve the dehydrogenation performance of MgH_2_. The small nanocrystals distributed in the composites are a way to facilitate hydrogen transport and improve the dehydrogenation performance, while M_2_C is only a catalyst and not a reactant; the multivalent M atoms on the surface act as intermediates for electron movement between H^−^ and Mg^2+^, making dehydrogenation easier. This also reveals the catalytic effect of 2D carbon materials with different metal sites on improving hydrogen storage of MgH_2_.

## Data Availability

The original contributions presented in the study are included in the article/supplementary material, further inquiries can be directed to the corresponding author.
